# Cervico-Mediastinal Emphysema Due to Panic Attack: A Case Report

**DOI:** 10.7759/cureus.100427

**Published:** 2025-12-30

**Authors:** Thomas P Clements, Antonia P Tse, Tom J Rourke

**Affiliations:** 1 Intensive Care Unit, Bristol Royal Infirmary, Bristol, GBR; 2 Department of Otolaryngology, Royal Berkshire Hospital, Reading, GBR

**Keywords:** mediastinal emphysema, panic disorders, retropharyngeal emphysema, subcutaneous emphysema, tissue space emphysema

## Abstract

Spontaneous cervical emphysema is an uncommon entity characterised by free air in the deep neck spaces without obvious cause. The differential diagnosis of cervical emphysema is broad, because the visceral space is contiguous with mediastinum and retroperitoneum. We present an unusual case of spontaneous cervico-mediastinal emphysema (CME) that occurred in an 18-year-old female after a panic attack. The presenting features included dyspnoea, chest pain and subcutaneous emphysema. She had no other sign of aerodigestive tract pathology, and history was negative for risk factors such as asthma, vomiting and recreational drug use. Computed tomography of the neck and thorax demonstrated retropharyngeal and visceral space emphysema extending through the mediastinum. No other abnormalities such as lung bullae were apparent, leading to a diagnosis of spontaneous CME. The patient was admitted for observation, treated with intravenous antibiotics, and made a full recovery.

This case demonstrates that cervical emphysema can occur as a result of panic disorder and consequent hyperventilation. Respiratory or gastrointestinal pathology is not a prerequisite for the occurrence of CME.

## Introduction

Cervical tissue space emphysema is classically associated with pharyngeal infection or disruption of a mucosal surface [1]. Spontaneous pneumomediastinum (SPM) is an underappreciated cause of cervical emphysema and often occurs in the absence of aerodigestive tract or lung pathology [2].

SPM is an uncommon diagnosis accounting for approximately 1 in 30,000 emergency department attendances [2]. Air tracking into the neck is common, with cervical subcutaneous emphysema reported in 66% of cases [3]. Although the clinical course of spontaneous cervical and mediastinal emphysema (CME) is usually benign [2,3], there is broad differential including life-threatening conditions such as oesophageal rupture. Accordingly, management strategies focus on the exclusion of underlying pathology [2,3].

We present a rare case of CME arising after a panic attack, followed by a review of its pathogenesis. To our knowledge, this is the first report of deep neck space and mediastinal emphysema occurring in this context.

## Case presentation

An 18-year-old female was referred by her general practitioner with a two-day history of dyspnoea, right-sided pleuritic chest pain and a feeling of “crystals” in her neck. She described an episode of hyperventilation during a panic attack three days previously. There was no recent history of sore throat, dysphagia, cough, emesis, or recreational drug use. Past medical history was unremarkable and negative for respiratory or gastrointestinal disease.

Physical examination demonstrated non-tender cutaneous crepitus over the right anterior and posterior triangles of the neck. Examination of the head and neck was otherwise unremarkable, with normal appearances of the pharynx and supraglottis on flexible nasendoscopy. Vital signs were within normal limits.

**Figure 1:**
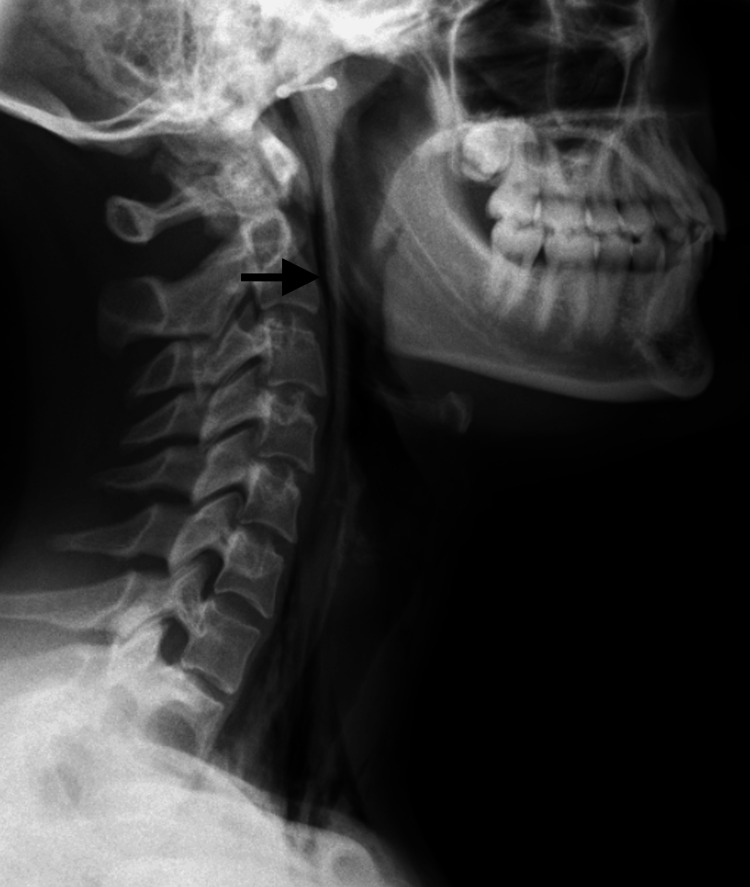
Lateral neck X-ray demonstrating retropharyngeal emphysema

Chest X-ray and routine laboratory investigations were unremarkable with a white cell count of 8.20 × 10^9^/l (reference range 4-11 × 10^9^/l) and C-reactive protein of 9.6 mg/l (reference range 0-5 mg/l). Lateral neck X-ray demonstrated retropharyngeal emphysema (RPE) (Figure 1).

**Figure 2:**
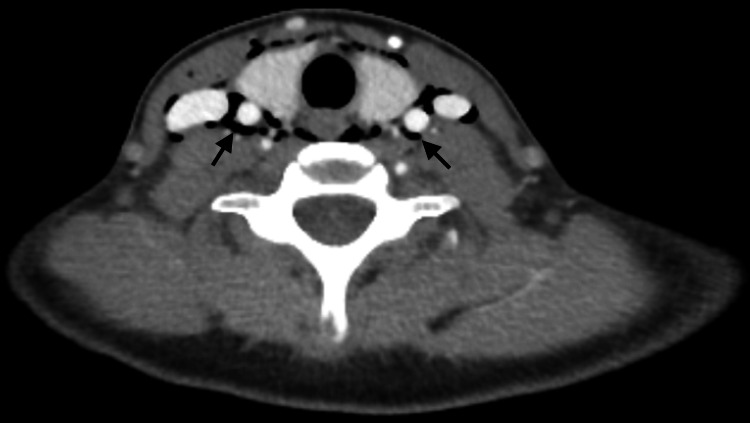
Computed tomography of the neck demonstrating extensive retropharyngeal, visceral, and carotid space emphysema

The patient was admitted for observation and treated with intravenous antibiotics to cover the risk of secondary infection (co-amoxiclav and metronidazole as per local protocols). Computed tomography (CT) of the neck and thorax demonstrated retropharyngeal and visceral space emphysema extending through the anterior and posterior mediastinum, terminating just inferior to the diaphragmatic hiatus (Figure 2). Subcutaneous emphysema was evident in the anterior neck and adjacent right chest wall overlying the pectoralis major. No other abnormalities, such as lung bullae, were apparent, leading to a diagnosis of spontaneous retropharyngeal and mediastinal emphysema.

Subcutaneous crepitation had improved significantly the day after presentation. The patient was converted to oral antibiotic therapy and discharged following an uneventful 48-hour admission. Outpatient review at two weeks demonstrated a full resolution of symptoms.

## Discussion

Tissue space emphysema may occur due to (i) an external penetrating injury, (ii) disruption of respiratory or gastrointestinal epithelia, or (iii) infection with gas-forming microorganisms such as *Clostridium perfringens*. Macklin deduced that SPM occurs due to alveolar rupture induced by excessive transmural pressures, with secondary movement of free air along bronchovascular sheaths to the hilum [4]. Once present in the mediastinum, free air may pass superiorly into the visceral compartment of the neck, inferiorly into the retroperitoneum, or superficially through the endothoracic fascia into the subcutis. Interstitial pressure gradients tend to favour movement in a vertical direction, meaning that the neck can be seen as a sentinel site for interstitial emphysema originating elsewhere.

SPM and RPE have been reported in association with a number of activities likely to cause abnormal transpulmonary pressures such as violent cough, emesis and straining [2,3,5]. Papadimos et al. reported a small pneumomediastinum around the left main bronchus following a panic attack without mention of cervical involvement [6]. The case at hand is the first report of deep neck space and retroperitoneal emphysema in the context of a panic attack and provides further evidence that psychogenic hyperventilation can result in extensive interstitial emphysema in the absence of aerodigestive tract or respiratory pathology. This concept is of relevance to emergency physicians assessing patients with panic disorder, who should consider and exclude pneumomediastinum in patients complaining of chest pain.

Spontaneous CME is usually a benign entity with a self-limiting course [2,3,5,7]. Uncommon complications of RPE include anterior displacement of the posterior pharyngeal wall with associated airway compromise [8,9]. Pneumomediastinum may cause pneumothorax due to rupture of the mediastinal pleura. Interstitial emphysema can become secondarily infected, and antibiotic therapy should be considered to prevent mediastinitis [10].

## Conclusions

Deep neck space emphysema is a poor localising sign and may reflect pathology in the mediastinum, pleural cavities or retroperitoneum. Assuming that the fascial layers of the neck are intact, the presence of air in multiple fascial compartments may suggest a common origin in the thorax. This case demonstrates that CME may occur as a result of panic disorder and consequent hyperventilation. Intrinsic respiratory or gastrointestinal pathology is not a prerequisite for the occurrence of CME. Spontaneous CME should be considered in all patients presenting with a panic attack and chest or neck pain.
